# From screen to structure with a harvestable microfluidic device

**DOI:** 10.1107/S1744309111024456

**Published:** 2011-07-26

**Authors:** Vivian Stojanoff, Jean Jakoncic, Deena A. Oren, V. Nagarajan, Jens-Christian Navarro Poulsen, Melanie A. Adams-Cioaba, Terese Bergfors, Morten O. A. Sommer

**Affiliations:** a National Synchrotron Light Source, Brookhaven National Laboratories, Upton, NY 11973, USA; b Structural Biology Resource Center, Rockefeller University, 1230 York Avenue, New York, NY 10065, USA; c JAN Scientific Inc., 4726 Eleventh Avenue NE, Suite 101, Seattle, WA 98105, USA; d Department of Chemistry, Biophysical Chemistry Group, University of Copenhagen, Universitetsparken 5, DK-2100 Copenhagen, Denmark; e Microlytic ApS, Universitetsparken 7, DK-4000 Roskilde, Denmark; f Department of Cell and Molecular Biology, Uppsala University, SE-75 124 Uppsala, Sweden

**Keywords:** Crystal Former, protein crystallization, structural biology, liquid–liquid diffusion, microfluidics

## Abstract

Advances in automation have facilitated the widespread adoption of high-throughput vapour-diffusion methods for initial crystallization screening. However, for many proteins, screening thousands of crystallization conditions fails to yield crystals of sufficient quality for structural characterization. Here, the rates of crystal identification for thaumatin, catalase and myoglobin using microfluidic Crystal Former devices and sitting-drop vapour-diffusion plates are compared. It is shown that the Crystal Former results in a greater number of identified initial crystallization conditions compared with vapour diffusion. Furthermore, crystals of thaumatin and lysozyme obtained in the Crystal Former were used directly for structure determination both *in situ* and upon harvesting and cryocooling. On the basis of these results, a crystallization strategy is proposed that uses multiple methods with distinct kinetic trajectories through the protein phase diagram to increase the output of crystallization pipelines.

## Abstract 2

Microfluidic crystallization using the Crystal Former improves the identification of initial crystallization conditions relative to screening *via* vapour diffusion.

## 1. Introduction   

Protein crystals are generated by perturbing the solubility of a con­centrated pure protein solution through the addition of precipitating reagents such as salts, polymers and other additives that promote crystal nucleation and growth (McPherson *et al.*, 1995 ▶). In addition to precipitant selection, which is critical to protein crystallization (Kimber *et al.*, 2003 ▶; Newman *et al.*, 2005 ▶; Page *et al.*, 2003 ▶), the method by which the precipitant is introduced to the protein solution also has a significant impact on crystal formation and quality (García-Ruiz, 2003 ▶; Gavira *et al.*, 2002 ▶). For example, convection-free environments, achieved under microgravity, within hydrogels or inside thin capillary tubes, promote the growth of highly ordered crystals of superior quality (Lorber *et al.*, 1999 ▶; Vergara *et al.*, 2005 ▶). Furthermore, fluid physics at the microscale allows gentle and well controlled diffusive mixing of solutions. Many studies have established that a diffusive mixing regime facilitates the crystallization process (Dhouib *et al.*, 2009 ▶; Emamzadah *et al.*, 2009 ▶; Hansen *et al.*, 2002 ▶; Ng *et al.*, 2003 ▶, 2008 ▶).

**Figure 1:**
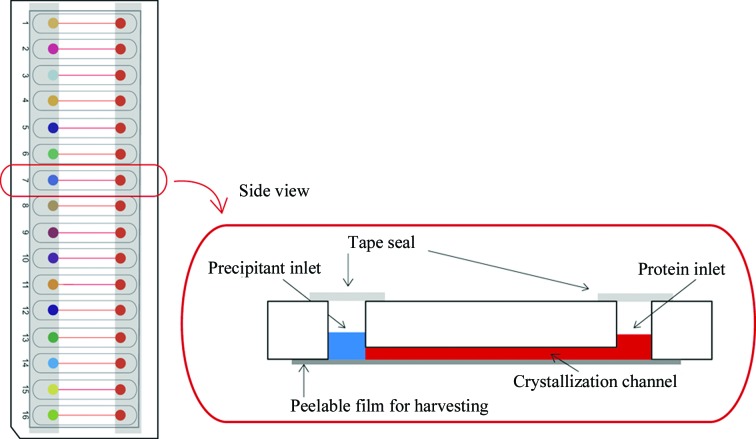
A microfluidic device comprised of 16 microchannels. Using conventional pipettes, the protein sample is loaded into one sample inlet and the crystallization solution is applied to the opposing channel. The inlets are sealed and the Crystal Former is incubated at the desired temperature. A thin removable sealing film forms the rear of each microchannel, permitting crystal access for harvesting and X-ray diffraction studies.

Capitalizing on the advantages of convection-free diffusion for protein crystallization, various microfluidic devices have been developed and shown to improve protein-crystallization output (Anderson *et al.*, 2007 ▶; Garcia-Ruíz *et al.*, 2002 ▶; Gavira *et al.*, 2002 ▶; Hansen *et al.*, 2004 ▶, 2006 ▶; Li *et al.*, 2010 ▶; Yadav *et al.*, 2005 ▶). In this study, we evaluate the impact of employing a commercially available microfluidic device, the Crystal Former (Fig. 1 ▶), on initial screening of several well characterized proteins. We have performed crystallization trials using the Crystal Former in parallel with vapour diffusion for the crystallization of thaumatin, catalase and myoglobin. Furthermore, we assess the validity of claims that crystals grown in this device can be harvested for data collection and yield crystals of high quality that can be used directly for structure determination. In doing so, we have determined the structure of thaumatin from crystals harvested from the Crystal Former and of lysozyme *via in situ* diffraction experiments.

## 2. Materials and metods   

### 2.1. Model protein preparation   

Thaumatin from *Thaumatococcus daniellii* (Sigma, catalog No. T7638) was dissolved in distilled water to 50 mg ml^−1^. Catalase and myoglobin (Sigma, USA) were solubilized in distilled water to 30 and 62 mg ml^−1^, respectively. Lysozyme was purchased from Hampton Research (catalog No. HR7-108) and was reconstituted to a final concentration of 20 mg ml^−1^ in distilled water.

### 2.2. Crystallization of thaumatin, catalase and myoglobin by sitting-drop vapour diffusion   

The crystallization conditions for thaumatin, catalase and myo­globin were determined using conditions 1–48 of the Crystal Screen (Hampton Research, USA) and 1–48 of the JCSG-plus (Molecular Dimensions, UK) sparse-matrix screens. For sitting-drop vapour diffusion, 0.5 µl protein solution was mixed with 0.5 µl crystallization solution and equilibrated against 100 µl of the crystallization condition in the reservoir. Sitting-drop plates were sealed with Crystal Clear tape (Hampton Research, USA) and incubated at room temperature for 7 d.

### 2.3. Crystallization of thaumatin, myoglobin and catalase by liquid–liquid diffusion   

The Crystal Formers (Microlytic; http://www.microlytic.com) used in this study each comprised 16 microchannels. Each channel is bounded by two sample-inlet wells. Protein sample was first applied and the channel was allowed to fill by capillary action. The precipitant solution was then applied to the opposing inlet and the experiment was sealed. No external equipment controls sample loading in this format. All three proteins were screened using the Crystal Former in parallel with vapour-diffusion trials. For these trials, 0.3 µl protein sample was applied to each channel followed by the loading of 0.3 µl crystallization reagent into the opposing inlet. The Crystal Formers were then sealed with the sealing tape provided and incubated at room temperature for 7 d. The crystallization trials were inspected manually.

### 2.4. Harvesting of thaumatin crystals from the Crystal Former   

Thaumatin crystals grown from Crystal Screen condition No. 29 (0.1 *M* HEPES pH 7.5, 0.8 *M* potassium sodium tartrate tetrahydrate) were selected for data collection. Access to the crystals was accomplished by scoring the sealing film on the back of the Crystal Former with a scalpel. Once exposed, 5 µl of cryoprotectant was added to the open channel to prevent drying of the crystal during manipulations and to protect the crystal during subsequent flash-cooling. Cryo­protectant solutions were generated by combining the respective crystallization condition with an equal volume of 50%(*v*/*v*) glycerol that had been prepared in distilled water. Thaumatin crystals were harvested using nylon loops (Hampton Research, USA). Crystals were flash-cooled and stored in liquid nitrogen for subsequent data collection. Diffraction properties were evaluated on the X6A beam­line at National Synchrotron Light Source (Brookhaven National Laboratory, Upton, New York, USA) and complete data sets were collected for suitable crystals.

### 2.5. Crystallization and *in situ* diffraction analysis of lysozyme crystals in the Crystal Former   

Given the improved crystallization hit rates obtained in the Crystal Former (Microlytic; http://www.microlytic.com), we wanted to evaluate whether the device was UV-compatible so that protein crystals formed in it could be detected *in situ* using a UV microscope (JAN Scientific, USA). The Crystal Former and the channels were imaged by placing the microfluidic device in a slide holder mounted on the *XY* stage of a UVEX microscope. Crystals were imaged using both the 5× and 15× objectives.

### 2.6. Data collection, processing and structure determination of thaumatin and lysozyme   

All data were collected at 100 K on the X6A beamline of the National Synchrotron Light Source (Brookhaven National Laboratory, Upton, New York, USA). Complete data sets each consisting of 300 frames of 1° oscillations were recorded using an ADSC Q210 detector. The exposure time was 30 s and the crystal-to-detector distance was 200 mm. Data processing and scaling was performed with *HKL*-3000 (Minor *et al.*, 2006 ▶). Solvent content was computed using the *CCP*4 package (Winn *et al.*, 2011 ▶; Matthews, 1968 ▶; Kantardjieff & Rupp, 2003 ▶). The structures were determined by molecular replacement using *MOLREP* (Vagin & Teplyakov, 2010 ▶). Refinement consisted of repeated cycles of model building in *Coot* (Emsley & Cowtan, 2004 ▶) and refinement in *REFMAC* (Murshudov *et al.*, 2011 ▶). The search models were PDB entries 2vi3 for thaumatin (Asherie *et al.*, 2009 ▶) and 2cgi for lysozyme (Jakoncic *et al.*, 2006 ▶).

## 3. Results   

### 3.1. Diffusive mixing kinetics increased crystallization productivity relative to vapour diffusion for well characterized proteins   

Thaumatin, catalase and myoglobin were screened with a subset of solutions from the sparse-matrix screens Crystal Screen (48 conditions) and JCSG-plus (48 conditions) in parallel in sitting-drop vapour-diffusion plates and Crystal Former devices. All three proteins yielded crystals in both methods. Remarkably, there were significant differences in the identities and numbers of crystallization con­ditions for the Crystal Former and sitting-drop experiments. For trials with the Crystal Former, crystals were obtained for 5, 28 and 8% of all conditions for thaumatin, catalase and myoglobin, respectively. The success rates were 1, 7 and 1% for thaumatin, catalase and myoglobin, respectively, using sitting-drop vapour diffusion. Consequently, a fourfold to eightfold increase in success for initial crystallization trials was observed in the Crystal Former (Table 1 ▶).

**Figure 2:**
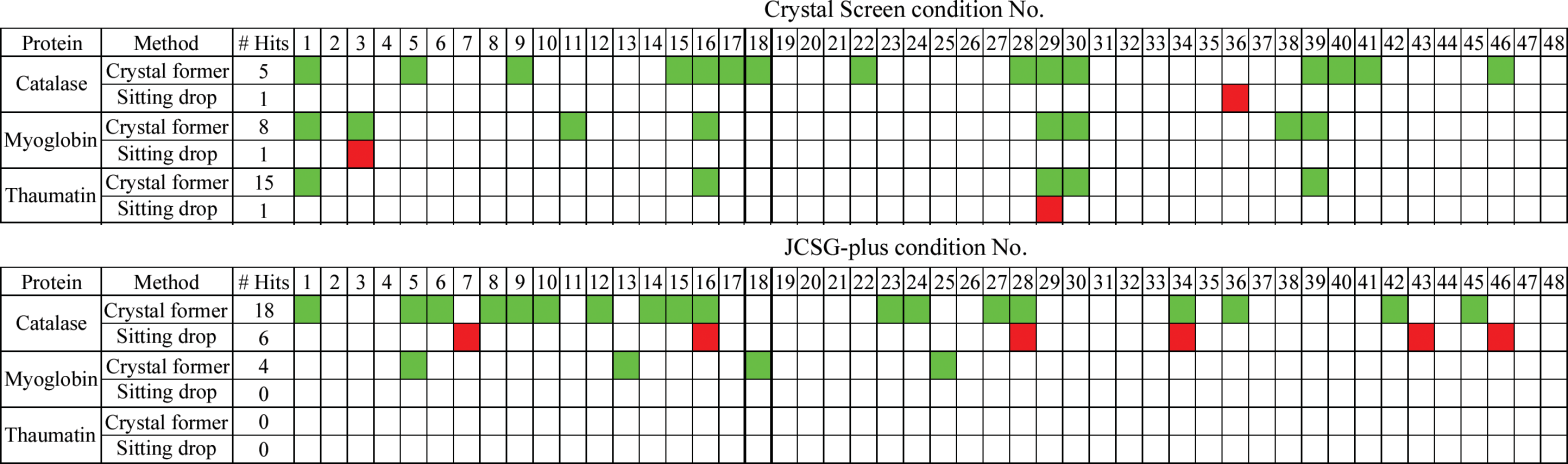
Each protein was screened by Crystal Screen (Hampton Research) and JCSG-plus 1 (Molecular Dimensions, UK) crystal screening using both sitting-drop vapour diffusion and the Crystal Former. Crystallization conditions identified with the Crystal Former are shown in green. Vapour-diffusion crystals are shown in red.

A comparison of the identified crystallization conditions revealed striking differences between vapour diffusion and the microfluidic device (Fig. 2 ▶). Approximately 40% of the conditions identified by vapour diffusion were unique to that method and were not captured in the Crystal Former trials. Similarly, 90% of the crystals grown in the Crystal Former were not identified by the sitting-drop experiments, highlighting the advantages of the Crystal Former in sampling the protein phase space.

To verify that the crystals obtained using the Crystal Former were indeed protein crystals, a representative subset was harvested and their respective diffraction was analyzed on the X6A beamline at the National Synchrotron Light Source (Brookhaven National Laboratory, Upton, New York. USA). For each mounted crystal, several frames were collected in order to verify that these were indeed protein crystals. The maximal resolution obtained from these crystals ranged from 2.8 to 1.1 Å, highlighting the good diffraction quality of the extracted crystals.

### 3.2. Harvesting and structure determination of thaumatin   

**Figure 3:**
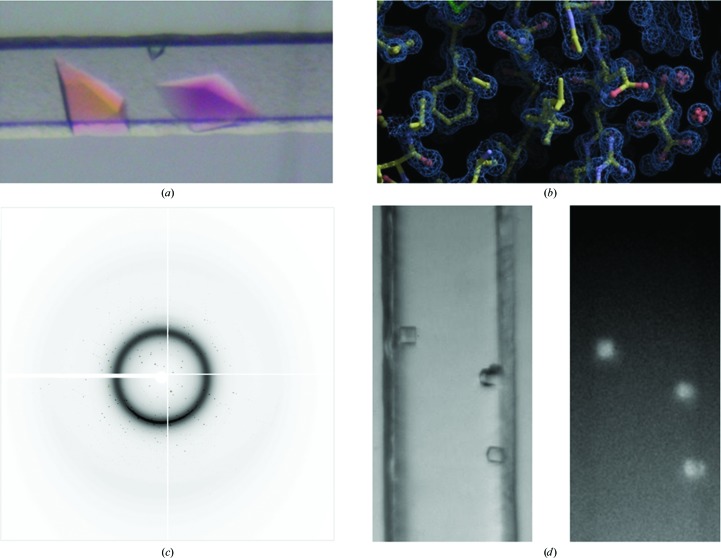
(*a*) Crystals of thaumatin were grown from 0.8 *M* potassium sodium tartrate, 0.1 *M* HEPES pH 7.5 in the Crystal Former. Here they are shown under polarized light. (*b*) Representative 2*F*_o_ − *F*_c_ electron density for the refined thaumatin structure at 1.25 Å resolution shown with 1.5σ contours. (*c*) The diffraction pattern of lysozyme crystals grown in the Crystal Former. X-ray data were collected *in situ* at room temperature on the X6A beamline (National Synchrotron Light Source, Brookhaven National Laboratory, Upton, New York, USA). (*d*) Detection of protein crystals in the Crystal Former using a UVEX microscope. Brightfield (left) and UV-fluorescence (right) images of lysozyme crystals within the microchannels of the Crystal Formers are shown. The microchannel width is 150 µm and the exposure lengths were 0.5 and 1 s for the brightfield and fluorescence images, respectively.

Thaumatin crystals were grown and prepared for data collection as described previously (Fig. 3 ▶ *a*). A single crystal was mounted in a cryoloop and cooled in liquid nitrogen. A complete set of diffraction data was collected and the structure was determined by molecular replacement (Fig. 3 ▶ *b*). Electron density was apparent for 206 of the 207 amino-acid residues. The thaumatin structure was refined to 1.25 Å resolution, with *R* and *R*_free_ values of 15.4% and 16.9%, respectively (Table 2 ▶). A single thaumatin monomer was modelled in the asymmetric unit, along with 201 water molecules and ten tartrate ions. This crystal form was isomorphous to 11 structures previously deposited in the Protein Data Bank (1lr3, 1rqw, 2blr, 2blu, 2d8p, 2g4y, 2oqn, 2vi2, 3dzp, 3dzr and 3e0a). For these depositions, the resolution ranged from 1.05 to 2.3 Å and the *R*_free_ values spanned the range 15.2–25%. As observed in the thaumatin structure reported here, no density was observed for the C-terminal alanine in PDB entries 2blr and 2blu. The remaining entries report unambiguous density for all 207 residues of thaumatin.

### 3.3. *In situ* data collection and structure determination for lysozyme   

The Crystal Formers were also assessed for their compatibility with *in situ* X-ray analysis on the X6A beamline (National Synchrotron Light Source, Brookhaven National Laboratory). The Crystal Former was mounted lengthwise with the microchannels perpendicular to the goniometric head. Not only could the Crystal Formers be mounted for the identification of protein crystals, but a complete data set for lysozyme crystals contained within the microchannels could be collected *in situ* at room temperature (Fig. 3 ▶ *c*, Table 2 ▶). The crystal structure of lysozyme was determined at 1.65 Å resolution with *R* and *R*_free_ values of 17.2% and 22.2% for the final refined structure. These crystals were isomorphous to 78 previous PDB entries. The resolution of previously deposited lysozyme structures ranged from 0.94 to 3.9 Å, with *R*_free_ values ranging from 14.5 to 32.3%.

### 3.4. Compatibility of the Crystal Former with *in situ* UV analysis   

A commonly used approach for imaging protein-crystallization experiments is UV fluorescence. The amino acid tryptophan emits light at approximately 360 nm when excited with light of 280 nm; hence, if a target protein contains the amino acid tryptophan it should be possible to distinguish target protein crystals from precipitant crystals based on fluorescence. Lysozyme crystals (10–100 µm) were grown in the Crystal Former and the channels were imaged by placing the microfluidic device in a slide holder mounted on the *XY* stage of a UVEX microscope (JAN Scientific, USA). Fluorescence from the larger crystals (>100 µm) could be visualized with the 5× objective, whereas the small crystals (∼10 µm) could only be seen with the 15× objective by virtue of the higher fluorescence excitation, collection efficiency and higher spatial resolution of the higher power objective (Fig. 3 ▶ *d*). The background signal and UV absorption from the material of the Crystal Former was sufficiently low that fluorescence from even the smallest crystals could be detected reliably.

## 4. Discussion   

In this study, we have explored the value of using an alternative method to vapour diffusion for initial crystallization screening, namely liquid–liquid diffusion using the Crystal Former. We have been able to identify crystallization conditions using both the vapour-diffusion and liquid–liquid diffusion methods, with more crystallization conditions resulting from the latter method for all proteins systematically sampled in this work. It should be noted that many of the conditions identified were unique to the respective method and were not captured by the alternate method (Fig. 2 ▶). Various studies of crystallization rates by vapour diffusion indicate that proteins that are crystallizable typically do so within a relatively small number of conditions (Kimber *et al.*, 2003 ▶; Page *et al.*, 2003 ▶). Further exploration of crystallization conditions using the same technique reaches a point of diminishing returns whereby further exploration of additional chemical conditions becomes less likely to yield crystals. The increased number of crystallization conditions identified in this study using the liquid–liquid diffusion method further underscores the important contribution that alternative kinetic trajectories through protein phase space have on the success of crystallization in a given condition. We propose that a more effective strategy for initial crystallization screening would thus be to explore the same chemical space using different crystallization methodologies.

A variety of crystallization formats, including liquid–liquid diffusion, offer unique kinetic trajectories through the protein phase diagram. A comprehensive screening approach that incorporates multiple crystallization formats would therefore be expected to promote the increased identification of crystallization conditions relative to single-technique approaches. Indeed, the data presented here revealed remarkable differences in crystallization behaviour for proteins screened in parallel using Crystal Formers and sitting-drop vapour diffusion. The unique sampling of the protein phase diagram for each crystallization method underlies the distinctive crystallization behaviour observed for these proteins. This resulted in significant differences in crystallization rates, with a pronounced increase in crystallization conditions identified in the Crystal Former trials with all other screening variables constant. The improved mixing kinetics and sampling of the protein phase space, coupled with the compatibility of the Crystal Former to most experimental setups, makes this device well suited as a standard approach to complement the current workflow of both academic and industrial crystallography laboratories.

## Supplementary Material

PDB reference: lysozyme, 3qy4

PDB reference: thaumatin, 3qy5

Structure factors: contains datablock(s) r3qy4sf. DOI: 10.1107/S1744309111024456/en54783qy4sup1.hkl

Structure factors: contains datablock(s) r3qy5sf. DOI: 10.1107/S1744309111024456/en54783qy5sup2.hkl

## Figures and Tables

**Table 1:** Relative increase in crystallization outcomes for catalase, myoglobin and thaumatin using the Crystal Former and vapour-diffusion methods

	No. of crystallization conditions		
Protein	Crystal Former	Vapor diffusion	Improvement (fold)
Catalase	28	7	4
Myoglobin	8	1	8
Thaumatin	5	1	5

**Table 2:** Data collection and structure refinement Values in parentheses are for the highest resolution shell.

	Thaumatin (in loop, 100K)	Lysozyme (in device, room temperature)
Data reduction		
Wavelength ()	0.9537	0.9793
Space group	*P*4_1_2_1_2	*P*4_3_2_1_2
Resolution ()	25.001.25 (1.271.25)	20.001.65 (1.681.65)
Unit-cell parameters ()	*a* = *b* = 57.91, *c* = 150.13	*a* = *b* = 79.15, *c* = 38.02
*I*/(*I*)	31.0 (1.8)	22.3 (2.0)
Completeness (%)	99.7 (95.9)	90.2 (94.3)
*R*_merge_ † (%)	5.3 (49.9)	5.9 (47.4)
Multiplicity	7.7 (3.6)	2.9 (2.8)
Mosaicity ()	0.21	0.15
Solvent content (%)	48	31
No. of frames	360	35
Oscillation per frame ()	0.3	1
Refinement		
Resolution ()	23.001.25 (1.281.25)	19.201.65 (1.691.65)
*R*_work_ ‡/*R*_free_ § (%)	15.4/16.9	17.2/22.2
No. of protein residues/atoms	206/1570	129/997
No. of tartrate atoms	10 [1 TAR¶]	0
No. of waters	201	101
Average *B* (^2^)	12.00	21.67
Protein only	10.99	20.29
Tartrate ion	8.89	
Solvent	19.98	36.5
R.m.s.d.††		
Bonds ()	0.012	0.015
Angles ()	1.461	1.665
PDB entry	3qy5	3qy4

† *R*_merge_ = 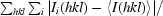 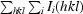, where *I_i_*(*hkl*) is the *i*th intensity measurement of reflection *hkl*, including symmetry-related reflections, and *I*(*hkl*) is its average.

‡ *R* = 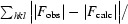 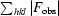, where *F*_obs_ and *F*_calc_ are the observed and calculated structure factors, respectively.

§ *R*_free_ was calculated using 5% of the diffraction data, selected at random, which were excluded from refinement.

¶ TAR refers to one tartrate ion.

†† Root-mean-square deviation.
